# Prognostic implications of a molecular classifier derived from whole‐exome sequencing in nasopharyngeal carcinoma

**DOI:** 10.1002/cam4.2146

**Published:** 2019-04-05

**Authors:** Hai‐Yun Wang, Fugen Li, Na Liu, Xiao‐Yun Liu, Xin‐Hua Yang, Yun‐Miao Guo, Jin‐Xin Bei, Yi‐Xin Zeng, Jian‐Yong Shao

**Affiliations:** ^1^ State Key Laboratory of Oncology in South China, Collaborative Innovation Center for Cancer Medicine, Guangdong Key Laboratory of Nasopharyngeal Carcinoma Diagnosis and Therapy Sun Yat‐sen University Cancer Center Guangzhou P. R. China; ^2^ Department of Molecular Diagnostics Sun Yat‐sen University Cancer Center Guangzhou P. R. China; ^3^ Research and Development Institute of Precision Medicine 3D Medicine Inc. Shanghai P. R. China; ^4^ BGI Genomics, BGI‐Shenzhen Shenzhen P. R. China; ^5^ Department of Experiment Research Sun Yat‐sen University Cancer Center Guangzhou P. R. China; ^6^ School of Laboratory Medicine Wannan Medical College Wuhu, Anhui Province P. R. China

**Keywords:** copy number variants, indels, molecular classifier, nasopharyngeal carcinoma, single‐nucleotide variants

## Abstract

The aim of this study was to use whole‐exome sequencing to derive a molecular classifier for nasopharyngeal carcinoma (NPC) and evaluate its clinical performance. We performed whole‐exome sequencing on 82 primary NPC tumors from Sun Yat‐sen University Cancer Center (Guangzhou cohort) to obtain somatic single‐nucleotide variants, indels, and copy number variants. A novel molecular classifier was then developed and validated in another NPC cohort (Hong Kong cohort, n = 99). Survival analysis was estimated by the Kaplan‐Meier method and compared using the log‐rank test. Cox proportional hazards model was adopted for univariate and multivariate analyses. We identified three prominent NPC genetic subtypes: RAS/PI3K/AKT (based on *RAS*, *AKT1*, and *PIK3CA* mutations), cell‐cycle (based on *CDKN2A*/*CDKN2B* deletions, and *CDKN1B* and *CCND1* amplifications), and unclassified (based on dominant mutations in epigenetic regulators, such as *KMT2C*/*2D*, or the Notch signaling pathway, such as *NOTCH1*/*2*). These subtypes differed in survival analysis, with good, intermediate, and poor progression‐free survival in the unclassified, cell‐cycle, and RAS/PI3K/AKT subgroups, respectively, among the Guangzhou, Hong Kong, and combined cohorts (n = 82, *P* = 0.0342; n = 99, *P* = 0.0372; and n = 181, *P* = 0.0023; log‐rank test). We have uncovered genetic subtypes of NPC with distinct mutations and/or copy number changes, reflecting discrete paths of NPC tumorigenesis and providing a roadmap for developing new prognostic biomarkers and targeted therapies.

## 1 INTRODUCTION

Nasopharyngeal carcinoma (NPC) has a unique geographical distribution that is distinct from other head and neck cancers. Currently, the tumor‐lymph node‐metastasis (TNM) staging system is the key clinical tool for prognostication, risk stratification, and making treatment decisions. Although patients of the same TNM stage receive similar treatments, their clinical outcomes vary greatly. Similarly, a previous study found comparable relative survival rates between undifferentiated and differentiated subtypes according to the World Health Organization histological classification.^1^ Thus, current staging and histological classification systems are insufficient for predicting patient survival. One current hypothesis is that differences in prognosis and treatment efficacy might be attributed to biological heterogeneity.

Proper classification is essential for physicians to properly assign treatments and evaluate clinical outcomes. In the past 2 decades, researchers have found gene expression profiling is altered between cancer patients and healthy controls.^2^, ^3^, ^4^ Despite its early promise as a diagnostic and prognostic tool, gene expression profiling remains cost‐prohibitive and challenging to implement in a clinical setting. In NPC patients, several studies have reported molecular classifications only based on the expression of protein‐coding genes and microRNAs (7‐9), which have not been widely applied in clinical settings, even though they are relevant to prognosis. Recently, molecular profiling has been achieved by high‐throughput analyses, making molecular classifications based on genetic lesions more comprehensive and prevalent in clinical cancer management. In colorectal cancer, *BRAF* V600E and activating *KRAS* mutations are associated with metastasis, leading to poor survival.^5^ The Cancer Genome Atlas has proposed a molecular classification mainly according to genomic mutations, amplifications, and fusion genes that divide gastric cancer into four subtypes, providing a roadmap for patient stratification and trials of targeted therapies.^6^ A large‐scale international database demonstrated that four consensus molecular subtypes with distinguishable features including mutations and copy number changes facilitated clinical treatment in colorectal cancer.^7^ Notably, the advent of large‐scale DNA molecular profiling has been helpful to identify novel molecular targets that can be applied to the treatment of particular cancer patients.^8^, ^9^ Thus, developing a molecular classifier from DNA high‐throughput sequencing data that can identify dysregulated pathways and candidate drivers in NPC is an urgent priority.

To gain further insight into the genetic heterogeneity of primary NPC and to establish a DNA‐based molecular classifier capable of performing multiple‐gene classification for prognostic and therapeutic stratification, we performed whole‐exome sequencing (WES) on 82 formalin‐fixed paraffin‐embedded NPC tumors as a discovery cohort (Guangzhou NPC Cohort [GZNPC]). The WES data of 99 external NPC cases (Hong Kong NPC Cohort [HKNPC]) were used as an independent validation cohort.^10^ This study revealed that a molecular classifier derived from somatic single nucleotide variants (SNVs), indels, and copy number variants (CNVs) could better illustrate NPC tumorigenesis, and thus, could be used as a tool to explore different therapeutic strategies.

## 2 MATERIALS AND METHODS

### 2.1 Clinical specimens

Between July 2007 and December 2012, we obtained 82 primary NPC tumor tissues and corresponding blood samples from the Department of Pathology and Biobank at Sun Yat‐sen University Cancer Center (GZNPC cohort, Guangzhou, China). All specimens were independently reviewed by two pathologists to determine World Health Organization histological classification and tumor cellularity. NPC specimens with >50% tumor cellularity were used for sectioning, nucleic acid extraction, and library preparation. Additionally, 99 NPC patients from a Hong Kong study were used as the validation cohort (HKNPC).^10^ Detailed clinical data of all patients are summarized in Table 1. This study was approved by the institutional review board of Sun Yat‐sen University Cancer Center (RDDA2019001009).

**Table 1:** Clinical characteristics of the surveyed NPC patients including 82 patients from Guangzhou and 99^a^ patients from Hong Kong, respectively

	Discovery cohort (n = 82, %)	External validation cohort (n = 99, %)	Total (n = 181, %)	*P* value
Age at diagnosis (years)				
Median	47	49	48	0.0721^b^
Range	19‐71	23‐80	19‐80	
OS, months				
Median	48	56	50	0.1029^b^
Range	7‐94	2‐122	2‐122	
PFS, months				
Median	31	22	29	0.3034^b^
Range	1‐63	2‐96	1‐96	
Sex				
Male	62 (75.6)	71 (73.2)	133 (74.3)	0.7346^c^
Female	20 (24.4)	26 (26.8)	46 (25.7)	
Unknown	0	2	2	
Smoking status				
Nonsmoker	45 (61.6)	47 (51.7)	92 (57.9)	0.0809^c^
Smoker	28 (38.4)	44 (48.3)	72 (42.1)	
Unknown	9	8	17	
Clinical stage				
Early stage (I + II)	7 (8.5)	25 (26.1)	32 (18.0)	0.0030^c^
Advanced stage (III + IV)	75 (91.5)	71 (73.9)	146 (82.0)	
Unknown	0	3	3	
WHO classification				
NKUC	73 (89.0)	91 (93.8)	164 (91.6)	0.0205^c^
NKDC	7 (8.5)	0	7 (3.3)	
KSCC	2 (2.5)	6 (6.2)	8 (5.1)	
Unknown	0	2	2	
PFS rate (5 year, 95% CI) (%)	45.4 (26.4‐62.7)	41.5 (30.1‐52.5)	46.4 (37.8‐54.5)	
OS rate (5 year, 95% CI) (%)	78.2 (63.5‐87.5)	65.9 (53.7‐75.5)	71.8 (62.9‐78.9)	

NPC, nasopharyngeal carcinoma; OS, overall survival; PFS, progression‐free survival; CI, confidence interval; NKUC, nonkeratinizing undifferentiated carcinoma; NKDC, nonkeratinizing differentiated carcinoma; KSCC, keratinizing squamous cell carcinoma.

a All NPC cases are from Asia.

b Wilcoxon rank sum test.

c Pearson's *x* ^2^‐test.

### 2.2 The whole‐exome sequencing

Details of DNA isolation methods are provided in the online Data S1. DNA library preparation for NPC tumors and matched controls was performed according to the Agilent SureSelect^XT^ protocol (Santa Clara, CA) with minor modifications. Briefly, 2 μg of tumor DNA and 200 ng of control DNA were fragmented by ultrasonication (M220; Covaris, Woburn, MA), and fragments were captured using SureSelect^XT^ Human All Exon V5+UTRs 75M (Cat no. 5190‐6214; Agilent). Quantities and sizes of the libraries were determined using a Qubit fluorescence detector (Life Technologies, Carlsbad, CA) and an Agilent Bioanalyzer 2100, respectively. Finally, WES libraries were hybridized to an Illumina HiSeq PE Cluster Kit and SBS kit v4 for enrichment and were sequenced by 150 paired‐end read lengths on an Illumina Hiseq 1500 sequencing platform including a dual eight‐base index barcode according to the manufacturer's protocols (Illumina, San Diego, CA). The WES raw sequence data reported have been deposited in the Genome Sequence Archive, Beijing Institute of Genomics (BIG), Chinese Academy of Sciences,^11^, ^12^ under accession number CRA001397 that are publicly accessible at http://bigd.big.ac.cn/gsa/s/29XtNNXW.

### 2.3 Variant calling

Analyses of WES data were performed to identify somatic alterations in each tumor, including somatic SNVs, indels, and CNVs. DNA from peripheral blood were used as references to filter germline variants. Procedures for generating *bam* files with preprocessing and detection of somatic SNVs and indels (≤50 bp) are described in the online Data S1. The detailed sequencing quality control of the tumors and corresponding germline DNA from blood are shown in the Figure S1. Potential pathogenic mutations were predicted by ParsSNP algorithm based on functional impact scores with default parameters.^13^ For pathway enrichment analysis, the web‐based Gene Set Analysis Toolkit (WebGestalt) was used. Multiple‐test‐correction *P* values were generated using the Benjamini–Hochberg method, and *q*‐values were used to rank significantly altered pathways. The CNVkit (v.0.7.12.dev0) was used for somatic CNV analysis.^14^ CNV status was calculated as the ratio of tumor read depth to the average read depth observed in a panel of normal samples with the CNVkit tool. To extract a set of high confidence CNVs, log 2 ratios were >0.8 for amplifications and <−0.8 for deletions. Mutation signature analyses were constructed using deconstructSigs algorithms with default parameters^15^ and were compared with the previous signatures in the Catalogue of Somatic Mutations in Cancer database.

### 2.4 Validation datasets

Mutation Annotation Format (MAF) files, including SNVs, indels, and CNVs derived from the 99 HKNPC tumors as an independent validation cohort^10^ were requested from Professor Peter S. Hammerman (Dana‐Farber Cancer Institute, Boston, MA) for the purpose of model validation and survival analysis. For tumor mutation burden (TMB) analysis, WES data of NPC patients from Singapore (n = 55)^16^ and Hong Kong (n = 49)^17^ were downloaded from Sequence Read Archive SRA035573 and SRA288429, respectively. The same bioinformatics pipeline was performed in the two sequencing datasets (see online Data S1).

### 2.5 Statistical analysis

The median follow‐up for GZNPC (n = 82) for overall survival (OS) and progression‐free survival (PFS) was 48 and 31 months (range: 7‐94 and 1‐63 months), respectively. Overall, 36 of 82 (43.9%) patients progressed, and 12 (14.6%) died. Survival analysis was conducted with the Kaplan–Meier method. OS and PFS probabilities were analyzed by either the log‐rank test or Cox proportional regression hazards models. All statistical analyses were performed using Stata 14.0 (Stata Corp., College Station, TX) and GraphPad Prism 5 (San Diego, CA). Comparisons of data between the enrolled groups were performed with the Wilcoxon rank‐sum test unless otherwise specified. The R package was used to perform correlations of genomic status and clinical features such as age, sex, and clinical stage. The Database for Annotation, Visualization and Integrated Discovery (DAVID) v6.8 was used for the genes with cellular functions and pathways analysis.^18^, ^19^ The threshold of the gene counts for enrollment was 3 with *P* value less than 0.05.

## 3 RESULTS

### 3.1 Potentially significant genes in NPC tumorigenesis

**Figure 1:**
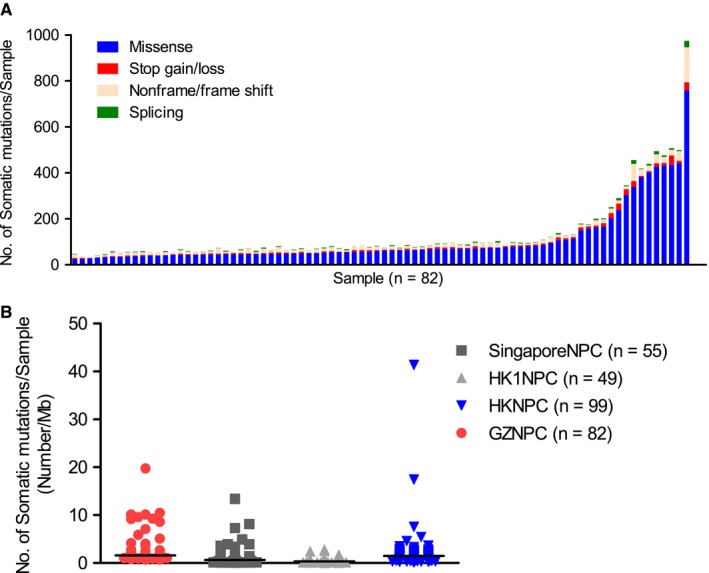
Somatic mutation types and distribution of mutation rates identified by whole‐exome sequencing among NPC cohorts. (A) Somatic mutation variants are profiled in each GZNPC patient (each column). (B) The tumor mutation burden (TMB) in GZNPC compared with the other three cohorts. Graphs show distributions of somatic TMB, defined as the number of nonsilent coding mutations per Mb. The black line indicates the threshold for samples with a median mutation burden in each NPC cohort.

To comprehensively profile somatic mutations, 82 NPC tumors and corresponding germline DNA were sequenced. Consequently, 10980 nonsilent somatic mutations in 6193 genes were discovered, including nonsynonymous SNVs, indels, stop gains/losses, and splicing mutations (Figure 1A and Table S1). We then compared TMB in GZNPC with those in the Singapore^16^ and Hong Kong cohorts,^10^, ^17^ with medians of 1.62, 0.64, 0.35, and 1.47, respectively. Difference was only found between GZNPC and the Singapore cohort (*P* = 0.0071, Figure 1B).

**Figure 2:**
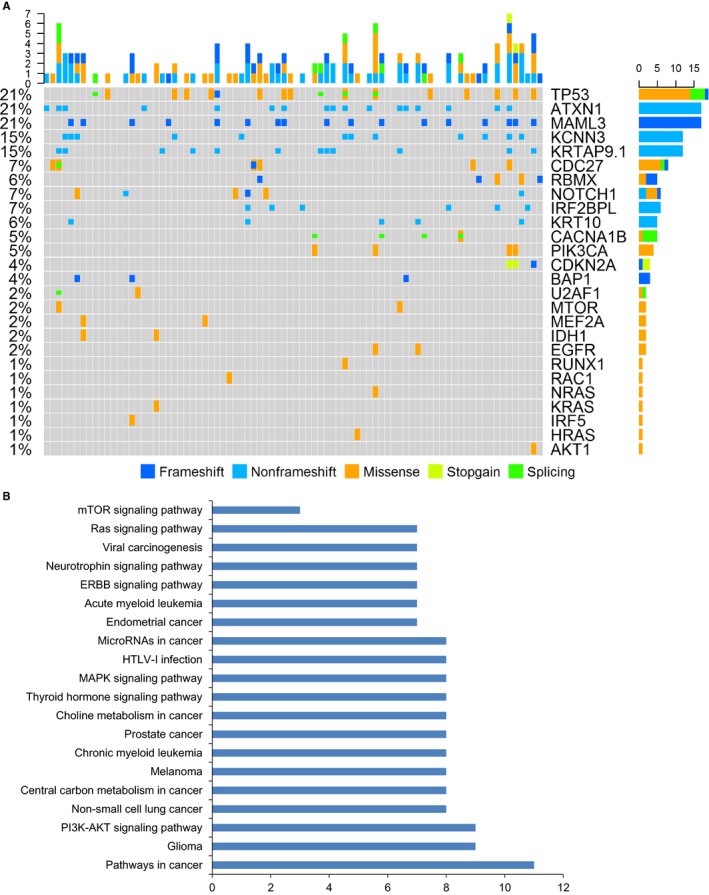
Important genes and the related functional and network analysis identified by ParsSNP and DAVID in 82 NPC patients. (A) A mutation matrix shows the important genes with ParsSNP score >0.1. The frequencies of gene mutations are plotted on the right. Variant classifications are displayed below. Columns indicate the examined cases, and rows indicate important genes. (B) All important genes are enriched into different cellular pathways by using the DAVID tool (*P* < 0.05). NPC, nasopharyngeal carcinoma; DAVID, The Database for Annotation, Visualization and Integrated Discovery.

We then applied ParsSNP to the GZNPC dataset of 10986 mutations. To narrow the focus, we only considered mutations with ParsSNP scores ≥0.07 (Table S2). This process readily included well‐studied cancer drivers such as *TP53*, *KRAS*, *NRAS*, *AKT1*, *BAP1*, and *PIK3CA*. Apart from these known cancer‐related genes, there were several new and important mutations that might affect NPC etiology and tumorigenesis (Figure 2A). We did the DAVID analysis for gene enrichments. Pathway in cancer, PI3K‐AKT, Ras, and ERBB signaling pathways are comprehensively existed in NPC patients (Figure 2B). Interestingly, we found *ATXN1* deletions (p.222_226del) with a ParsSNP score >0.1 in 15 NPC patients. The functional role of this mutation in NPC will require further study. The *U2AF1* mutation (exon3: p.R53C, ParsSNP score = 0.59) that was discovered in one case was previously reported to be a somatic mutation in colorectal cancer.^20^ We also found an oncogenic *IDH1* mutation (exon6: p.M182V, ParsSNP score = 0.52), which is rare in head and neck cancers but frequently occurs in glioma^21^ and cholangiocarcinoma, where mutated *IDH1* is associated with insensitivity to histone deacetylase inhibitors, irrespective of the specific mutation.^22^

A prominently altered pathway in NPC is the MAPK signaling pathway (*EGFR, NRAS, MEF2A, HRAS, KRAS, RAC1*, *P* < 0.001, Figure 2B), which was also observed in our study, but only partially in agreement with a previous study.^10^ A “hot spot” mutation in *HRAS* (exon3: p.Q61R, ParsSNP score = 0.48) was observed in one case. This was confirmed to be only present in metastatic sites of oral carcinoma and sensitive to BEZ‐235 (dual PI3K/mTOR inhibitor) and trametinib (MEK1/2 inhibitor), as reported by Akiyama *et al*.^23^ Finally, an *RAC1* mutation (exon3: R68H) that was identified to be pathogenic in colon cancer^24^ was detected in one NPC case.

### 3.2 Chromosome instability in NPC

**Figure 3:**
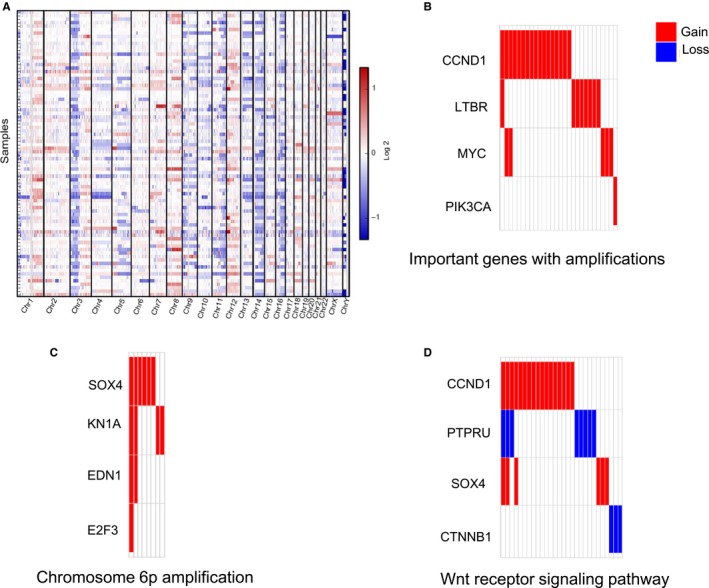
A global view of somatic CNVs and enrichment analysis of recurrent CNV changes involving the indicated genes in 82 NPC patients. (A) An overview of global chromosomal alterations with gain (red) and loss (blue) that were profiled in our cohort. Columns indicate recurrent arm‐level events, and rows indicate samples. (B) Amplifications of important oncogenes were frequently present in NPC patients. (C) Chromosome 6p amplification involving the indicated four genes was newly observed in NPC patients. (D) Amplifications/deletions of important genes in the Wnt signaling pathway in NPC are depicted. NPC, nasopharyngeal carcinoma; CNVs, copy number variants

Chromosomal alterations were predominated by large‐scale chromosomal arms with gains/losses, such as chr1p, chr1q, chr3p, chr3q, chr9p, and chr11q (Figure 3A and Table S3), which is similar to findings from previous studies.^25^

Several well‐known oncogenes that are activated in NPC, such as *CCND1* (11q13.3, 20.7%, 17/82), *LTBR* (12p13.31, 9.7%, 8/82), and *MYC* (8q24.12‐q24.13, 6.0%, 5/82) were recurrently amplified (Figure 3B) in GZNPC. High rates of chromosome 6p amplification, which include *SOX4* (6p22.3), *CDKN1A* (6p21.31), *EDN1* (6p24.1), and *E2F3* (6p22.3), are associated with cell proliferation (Figure 3C). Notably, six NPC patients with lymph node metastasis harbored *SOX4* amplifications, which is involved in the Wnt signaling pathway (Figure 3D) and consistent with a previous study,^26^ demonstrating that *SOX4* amplification was associated with lymph node metastasis and highly enriched in advanced‐stage NPC.

Several NPC patients in this cohort had deletions in tumor suppressor genes. A recurrent loss of *ADAMTS9* (3p14.1) was found in our cohort, consistent with previous fluorescence in situ hybridization findings.^27^ Arm‐level losses of *FHIT* (3p14.1‐p14.3) and other NPC‐related tumor suppressor genes^28^ were discovered in six NPC patients (6/82, 7.3%) and are concordant with previous findings.^10^ Recurrent *PTPRG* and *HepaCAM* deletions were newly detected in NPC patients (3p14, 7.3%, 6/82 and 11q24.1‐q24.2, 11.0%, 9/82, respectively). In this cohort, the most frequent deletion occurred in the chr9p21 region, spanning *CDKN2A* and *CDKN2B* (23.2%, 19/82 and 19.5%, 16/82, respectively). Chr13q deletions were frequently found in these 82 NPC patients and had previously been identified as allelic loss in a few studies.^29^, ^30^

### 3.3 A molecular prognosis classifier based on SNVs, indels, and CNVs

**Figure 4:**
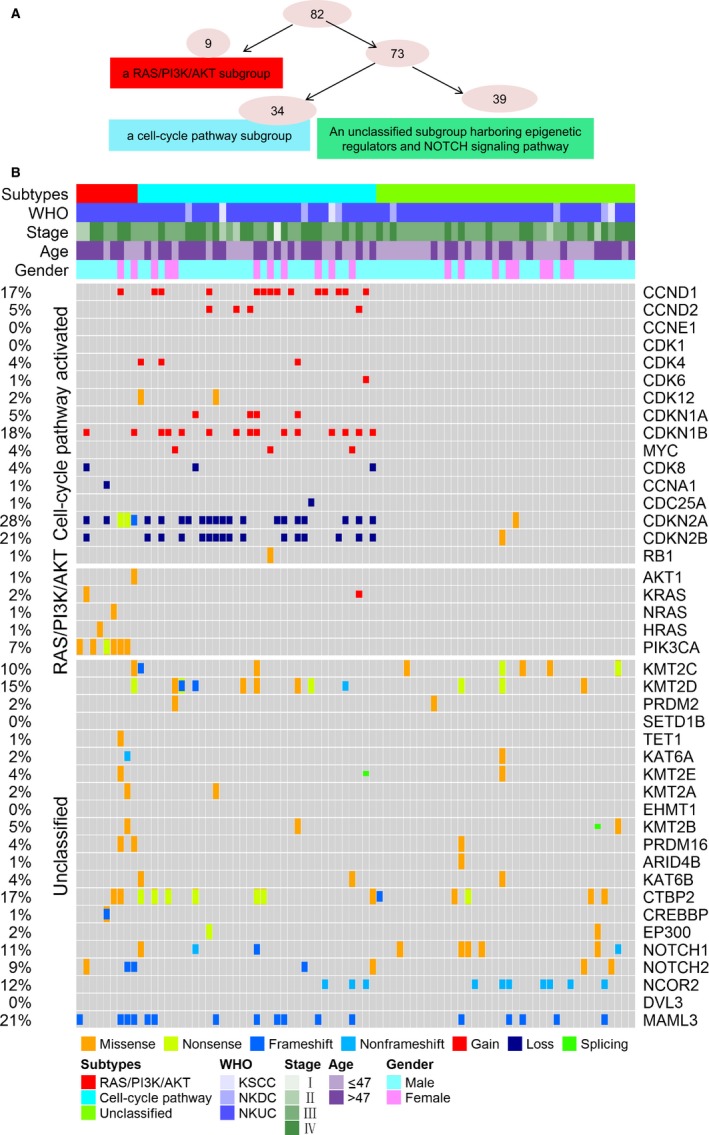
The proposed molecular classifier for 82 NPC patients. (A) A flowchart outlining how the 82 primary NPCs were orderly categorized into the molecular classifier. (B) NPCs were divided into three subtypes: the RAS/PI3K/AKT subgroup (red), the cell‐cycle subgroup (blue), and the unclassified subgroup (altered epigenetic regulators and Notch signaling pathway; green). Clinical features are depicted on the top. Columns indicate molecular events involving the molecular subtypes, and rows indicate samples enrolled in the analysis. NPC, nasopharyngeal carcinoma

The comprehensive analysis had been done for the genomic landscapes of NPCs in the previous studies and our own cohort, and the review of the biological functions associated with NPCs has also been carried out.^10^, ^14^, ^15^ According to the prior biological knowledge and the most enriched pathways, we proposed a decision tree based on somatic variants from the WES data that initially grouped the 82 NPC tumors into three subtypes (Figure 4A): (a) an RAS/PI3K/AKT subgroup (9/82, 10.9%) with *RAS*, *AKT1* and *PIK3CA* mutations, (b) a cell‐cycle subgroup (34/82, 41.5%) with high rates of *CDKN2A*/*CDKN2B* deletions and *CDKN1B* and *CCND1* amplifications, and (c) an unclassified subgroup (39/82, 47.6%) with dominant mutations in epigenetic regulators and the Notch signaling pathway. In the unclassified subgroup, apart from previously reported mutations in epigenetic regulators in multiple cancers, including *KMT2C*, *KMT2D*, *KMT2B*, *PRDM2*, *NCOR2*, *KAT6B,* and *EP300*, in this cohort, we also found new recurrent mutations in epigenetic regulators, including *CREBBP* and *PRDM16* (Figure 4B). The classifier was further validated in an external validation cohort (n = 99) and the combined cohorts (n = 181) (Figures S2 and S3).

**Figure 5:**
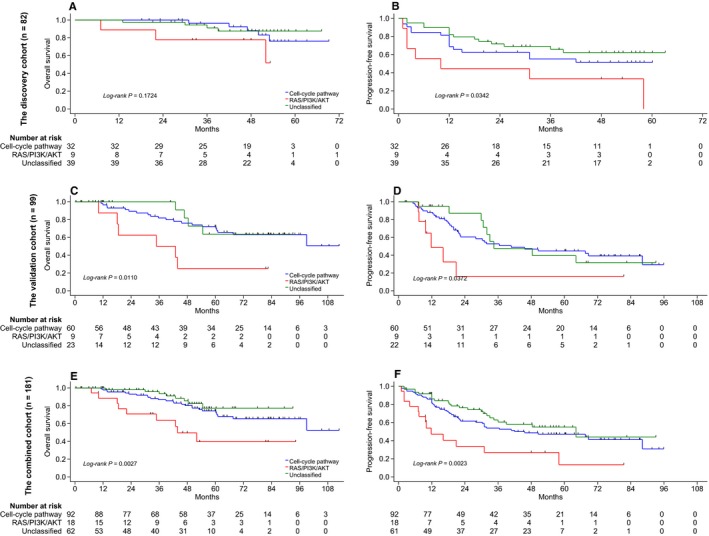
Kaplan–Meier OS and PFS curves stratified by the molecular classifier. OS analyses were performed in 82 (A, training cohort, *P* = 0.1724), 99 (C, external validation cohort, *P* = 0.0110), and 181 (E, combined, *P* = 0.0027) patients. Corresponding PFS analyses were separately conducted in 82 (B, *P* = 0.0342), 99 (D, *P* = 0.0372), and 181 (F, *P* = 0.0023) patients. The log‐rank test was used to estimate *P* values. OS, overall survival; PFS, progression‐free survival; NPC, nasopharyngeal carcinoma

To assess associations between the classifier and clinical phenotypes, a Kaplan–Meier survival analysis of the three subtypes was performed for GZNPC (*P* = 0.1724 for OS, Figure 5A; *P* = 0.0342 for PFS, Figure 5B, log‐rank test). The PFS of NPC patients among the three subtypes were significantly different (*P* = 0.0372, Figure 5D; *P* = 0.0023, Figure 5F). Additionally, to estimate the contribution of the classifier to survival, we performed univariate and multivariate COX regression analyses on the combined dataset. These showed that NPC patients in the RAS/PI3K/AKT subgroup were significantly associated with poorer PFS and OS in both univariate and multivariate analysis, while the cell‐cycle subgroup was not (Tables S4 and S5).

Combined analysis of clinical features revealed that the cell‐cycle (53.0%, 87/164) and unclassified (36.6%, 60/164) subgroups were primarily enriched in nonkeratinizing and undifferentiated carcinomas (Figure S4A). We estimated that the cell‐cycle subgroup comprised 54.9% of male cases (Figure S4B). We did not observe any systematic differences in age distribution among the three subtypes (Figure S4C).

## 4 DISCUSSION

Nasopharyngeal carcinoma is a highly heterogeneous malignancy with various outcomes even among patients with the same clinical stages and/or histopathological classifications. This study proposed three novel NPC subcategories based on gene sets with somatic SNVs, indels, and CNVs: (a) an unclassified subgroup, (b) a cell‐cycle subgroup, and (c) a RAS/PI3K/AKT subgroup, revealing that different mutagenic processes are operative through NPC development.

Abnormalities in the RAS/PI3K/AKT and cell cycle pathways result in dysregulated kinase activity and malignant transformation. Recent studies have shown good responses to a selective pan‐AKT inhibitor in phase I/II open‐label study in advanced solid malignancies, including breast and gynecologic cancers, with *PIK3CA* mutations.^31^, ^32^ Another investigation demonstrated the potential promise of combined MEK and MDM2 inhibitors for treating *KRAS*‐mutant nonsmall cell lung cancer and colorectal cancer.^33^

“Copy‐number‐driven” findings have been described in ovarian, breast, and lung cancer, and frequently recurrent amplifications and deletions of oncogenes and tumor suppressor genes, respectively, were observed in NPC. Frequent amplifications of cell cycle genes (*CCND1* and *CDK4*/*6*) suggest the potential of therapeutic inhibition of cyclin‐dependent kinases. Several studies have shown that cell lines with elevated *CCND1* and decreased *CDKN2A* (p16) expression are the most sensitive to CDK4/6 inhibitors.^34^ PD‐0332991, a selective CDK4/6 inhibitor, is being tested in advanced breast cancer with *CCND1* amplification and/or *CDKN2A* (p16) loss. We observed that the majority of NPC patients harbored loss of *CDKN2A*/*2B* and/or *CCND1* amplification, suggesting this subset of NPC patients could benefit from CDK4/6 inhibitors.

Epigenetic regulators are frequently mutated in cancers, including NPC.^17^ These genes were mostly present in all three subtypes. Accordingly, these altered epigenetic genes could be considered common molecular features of NPC. The epigenetic regulators that were newly found with recurrent mutations in our cohort, including *CREBBP* and *PRDM2*, have been separately identified as epigenetic regulators that could be targets in juvenile myelomonocytic leukemia^35^ and lung cancer.^36^ Further studies will be needed to investigate the functional roles of these genes in NPC. We also found additional noteworthy pathways enriched in NPC, such as pathways in cancer, ERBB, PI3K, Ras, and mTOR signaling pathway by using the DAVID tool, that are frequently targeted by genetic abnormalities in NPC. ERBB‐PI3K signaling pathway affected important cellular processes in NPC that was partially consistent with Lin et al' s study.^16^ Furthermore, AKT1, TP53, RAS, mTOR, and PIK3CA were recurrently present in the pathways indicated above, which were concordant with previous studies.^10^, ^16^, ^17^

Previous studies have reported that aberrant activation of the Notch signaling pathway can be detected in the majority of NPC samples and that inhibition of Notch signaling pathway enhances sensitivity to cisplatin/radiotherapy in EBV‐positive NPC patients and cell lines.^37^, ^38^, ^39^ Together, these genes could be potential cancer‐drivers and therapeutic targets in NPC. Functional studies (in vitro and in vivo) are warranted, and a basket trial based on driver mutations could be possible.^40^

Tumor mutation burden is an emerging biomarker to predict sensitivity to immune checkpoint inhibitors and has been demonstrated to be associated with response to anti‐PD‐1/PD‐L1 immunotherapy.^41^ In this study, we performed a TMB comparison in our cohort vs other NPC cohorts and found that only minor differences in TMB existed among the cohorts. A higher sequencing depth for all exons was more likely to explain the subtle differences, even though the same bioinformatics pipeline was used. Importantly, the high TMB in the cohort is most likely attributed to inherent genomic alternations in the tumor. For HKNPC, tumor contents of enrolled samples were >30%, while the tumor contents in our cohort were >50%. This might also explain TMB differences among the cohorts. It has been shown in several malignancies, including lung cancer, renal cell carcinoma, and melanoma, that higher TMB is associated with responses to PD‐1/PD‐L1 inhibitors, but no such clinical trials have been conducted in NPC. More clinical trials are needed to confirm if NPC patients with higher TMB would benefit from immunotherapy.

This study provided the descripted genomic alterations in NPC patients in Guangzhou; further evaluated the clinical applications of the molecular classifier that derived from the high‐throughput tumor sequencing. The newly found genes mutations and CNVs required deeper investigation and additional systematic studies to fully assess the clinical cancer genomics long‐term effects on patients' outcome. The continue studies include a more detailed, longitudinal follow‐up. Additionally, tumor profiling data sharing within those incorporate laboratories and institutions engaged is essential to fully functionalize the potential across multiple centers in Guangzhou, to deeper mining their existing data sets.

It is important to note that the sample size of this study was relatively small, limiting the resolution power. Ideally, larger cohorts can be assembled to validate the findings of this study and make this classifier more detailed. Furthermore, prospective trials should be designed to determine whether the subgroups specified herein predict activity of corresponding targeted therapies.

In conclusion, we proposed a novel classifier based on somatic SNVs, indels, and CNVs in NPC patients that showed prognostic value. Importantly, our findings provide a clinical classification system that can be therapeutically exploited. Each NPC patient subgroup has distinct features that may be associated with sensitivities to targeted agents; thus, specific treatments for each subgroup may improve clinical outcomes.

## CONFLICT OF INTERESTS

The authors declare that they have no competing interests.

## AUTHORS' CONTRIBUTIONS

JYS and YXZ designed the study and interpreted data. HYW and FL wrote the manuscript and interpreted data. XYL, XHY, YMG, HYW, and NL analyzed WES data. HYW collected samples and conducted WES. JXB interpreted data. All authors read and approved the final manuscript.
