# Supplementary figures and images for: Prognostic implications of a molecular classifier derived from whole‐exome sequencing in nasopharyngeal carcinoma
Source: Cancer Med. 2019 Apr 5;8(6):2705–16. doi: 10.1002/cam4.2146 (PMC6558473; doi:10.1002/cam4.2146)

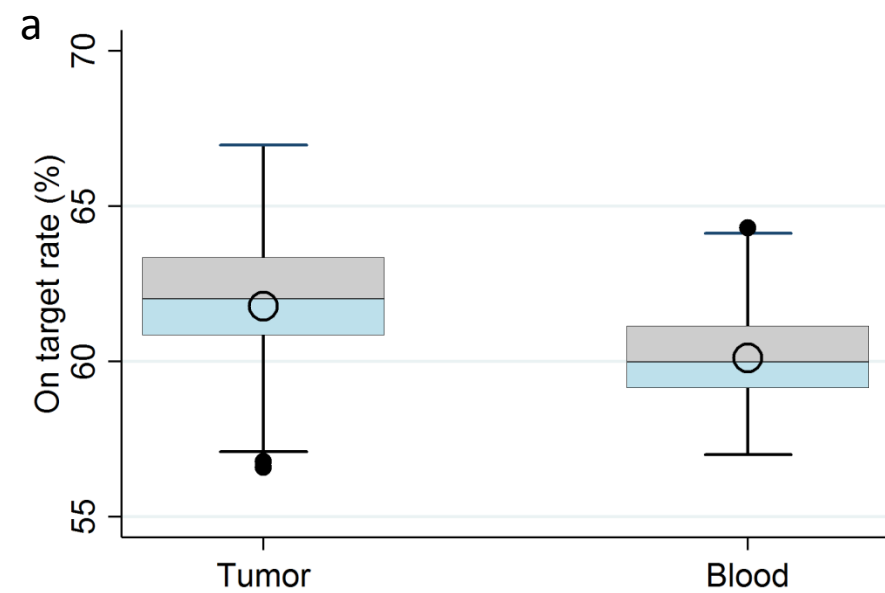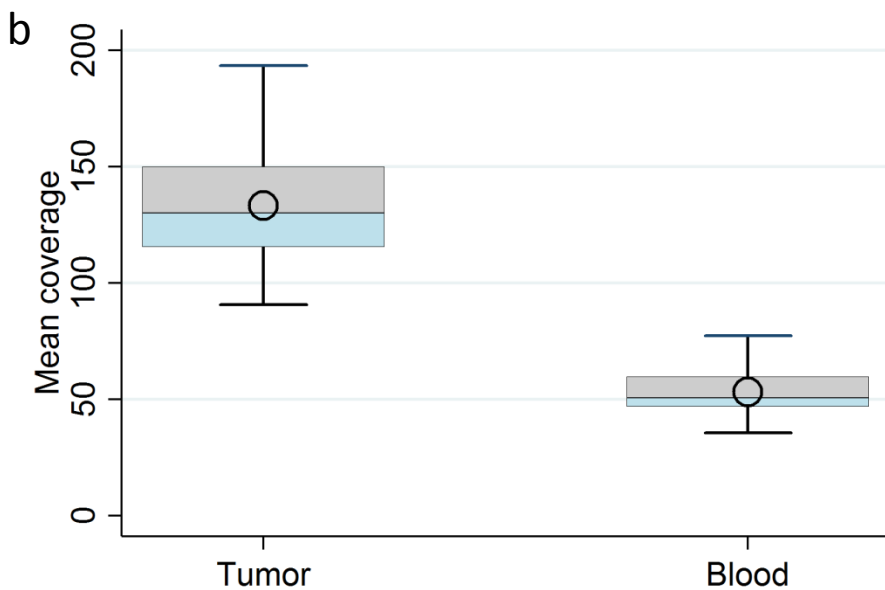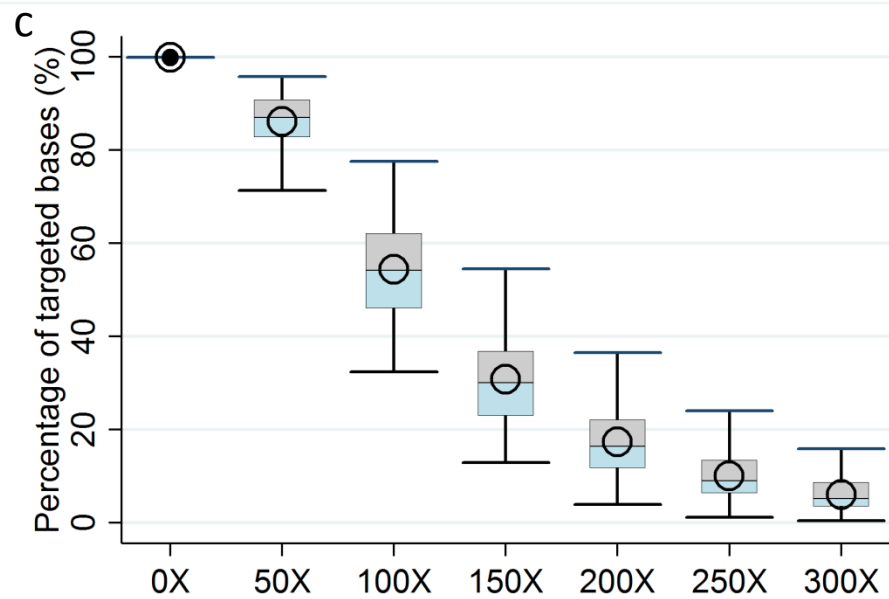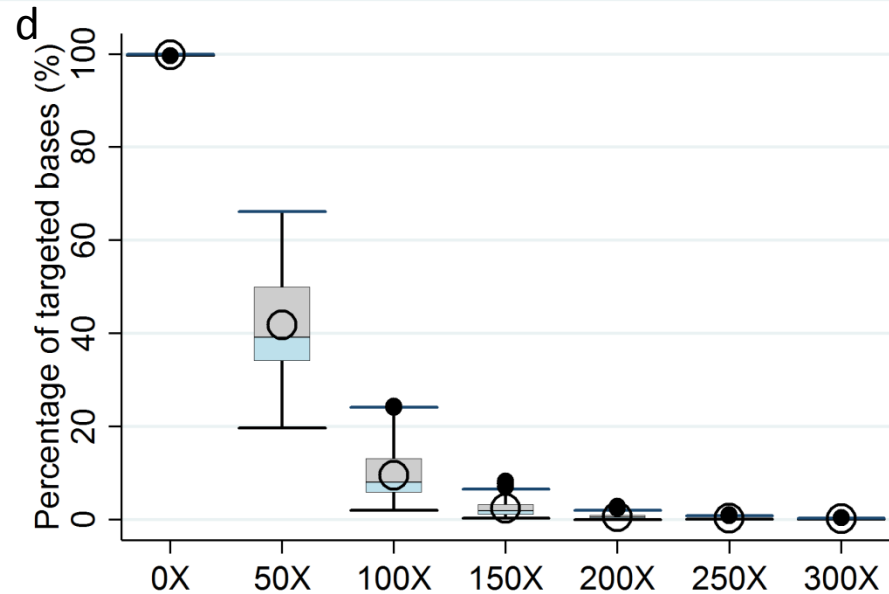

Supplement: Supplementary file 1 [file CAM4-8-2705-s001.pdf]

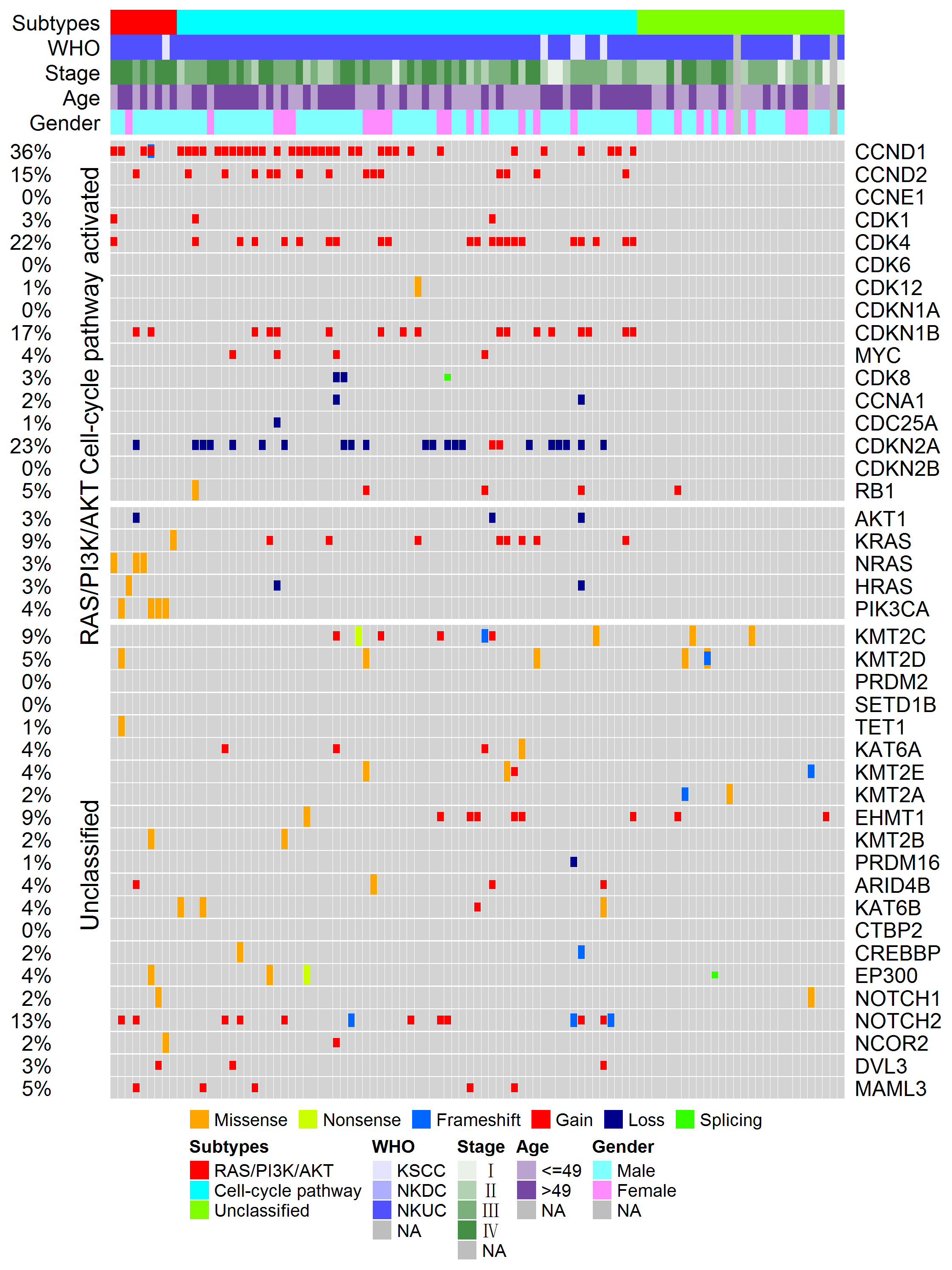

Supplement: Supplementary file 2 [file CAM4-8-2705-s002.tiff]

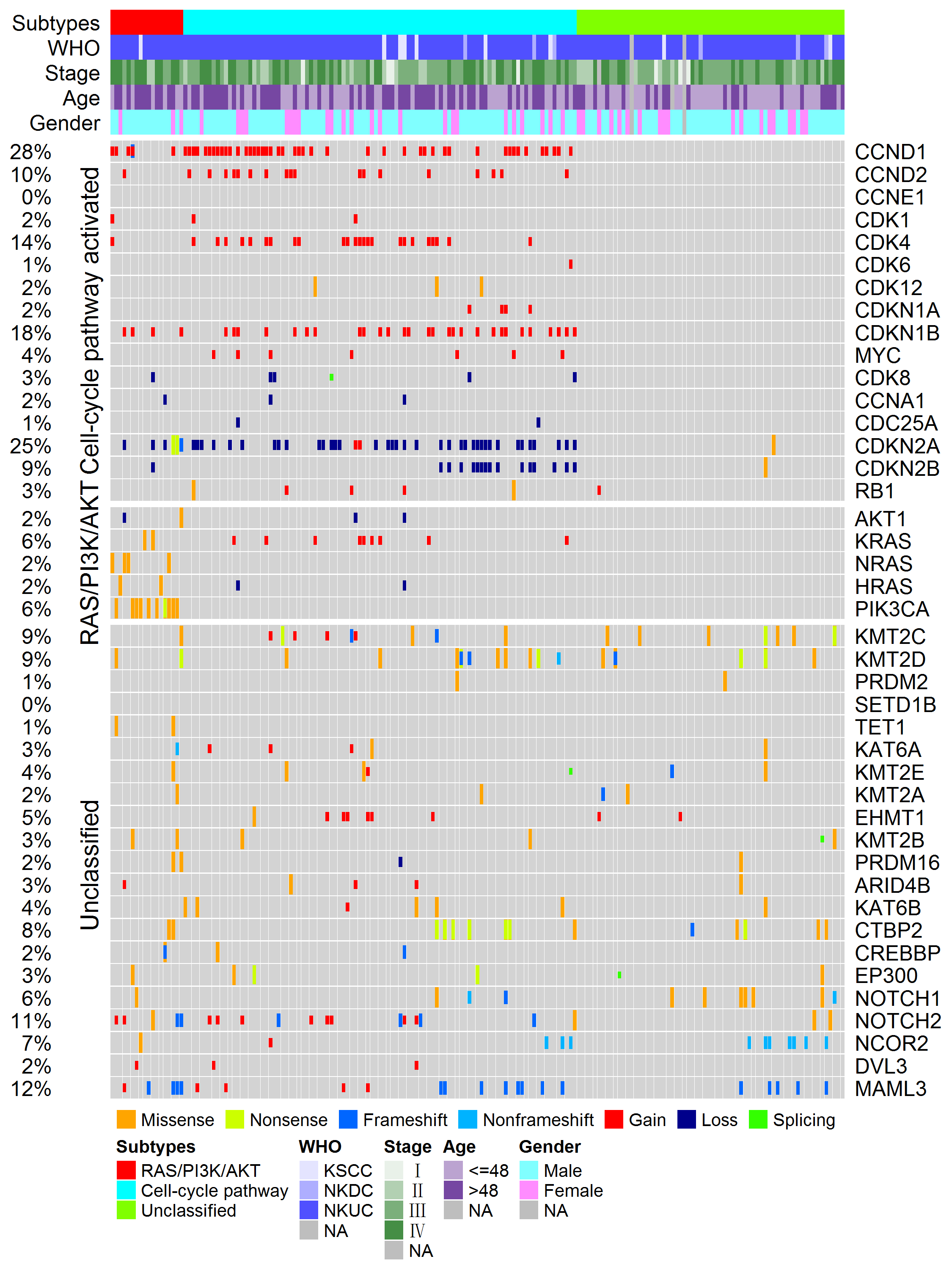

Supplement: Supplementary file 3 [file CAM4-8-2705-s003.tiff]

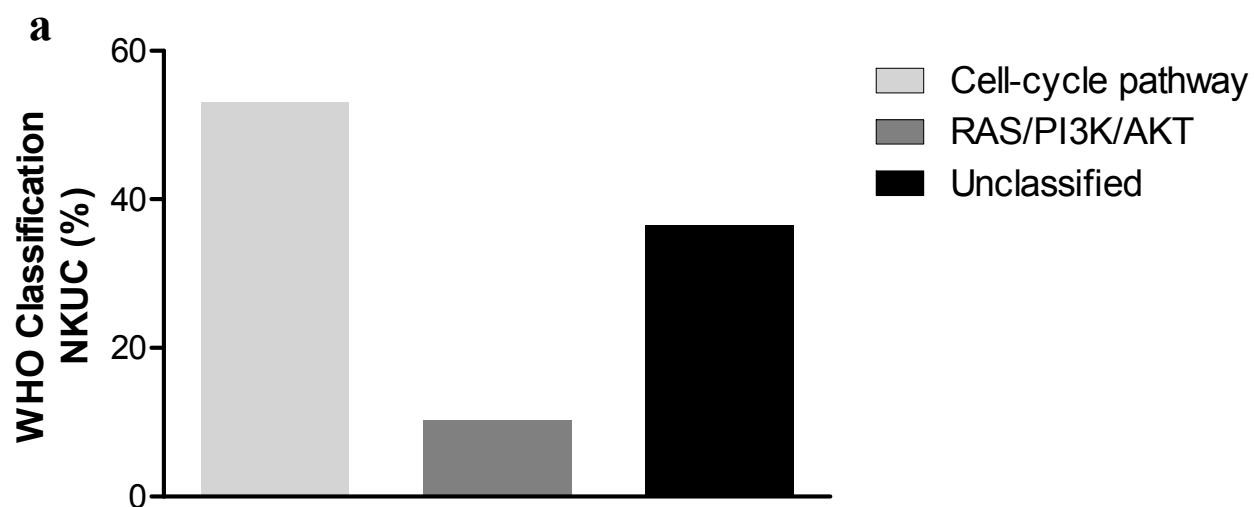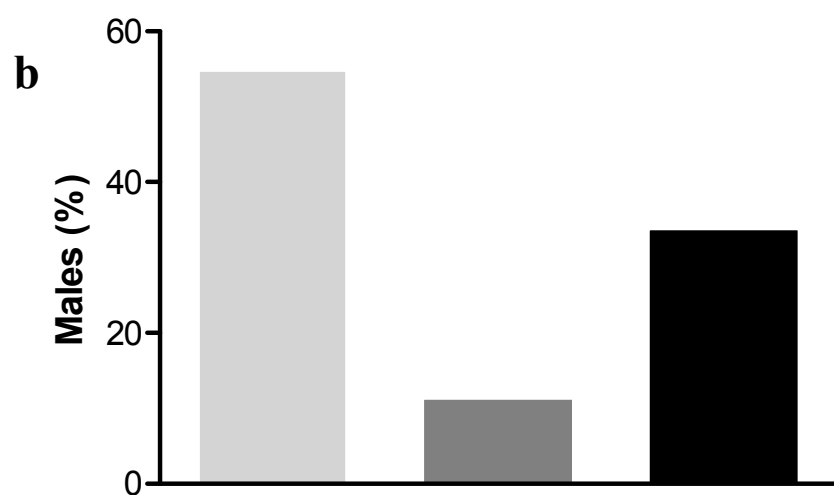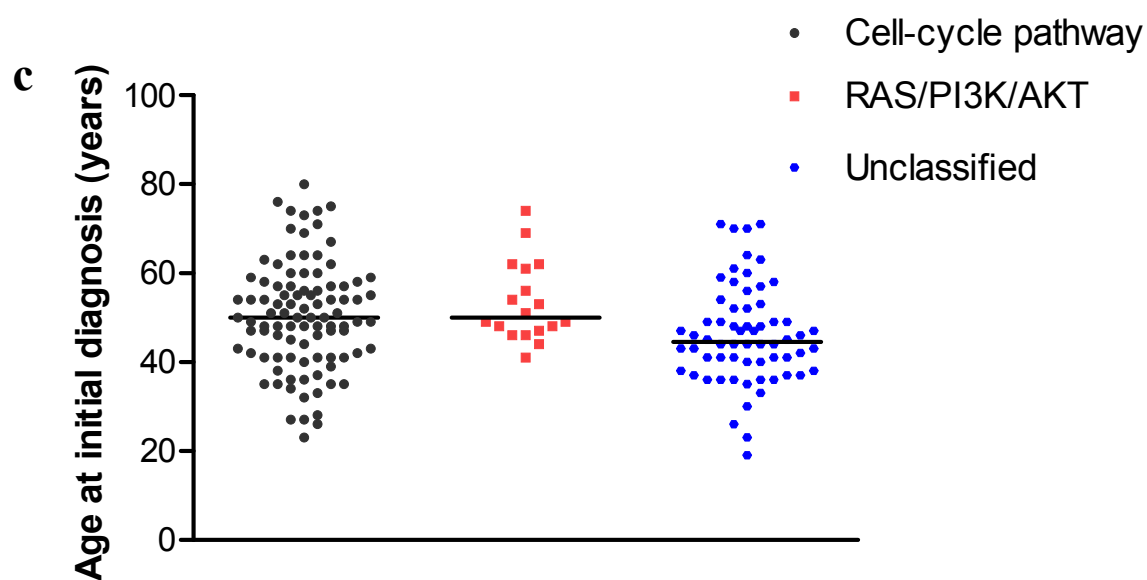

Supplement: Supplementary file 4 [file CAM4-8-2705-s004.pdf]

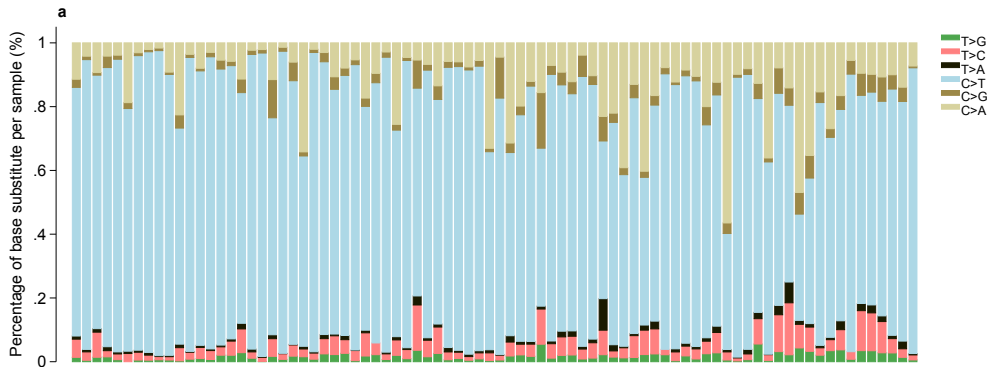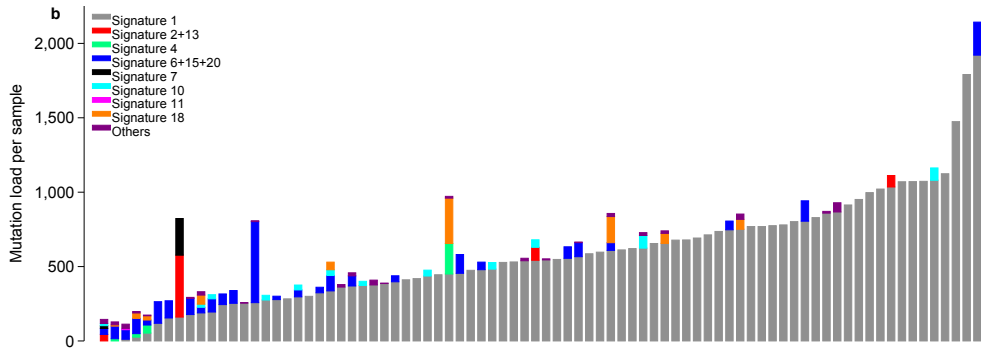

Supplement: Supplementary file 5 [file CAM4-8-2705-s005.pdf]

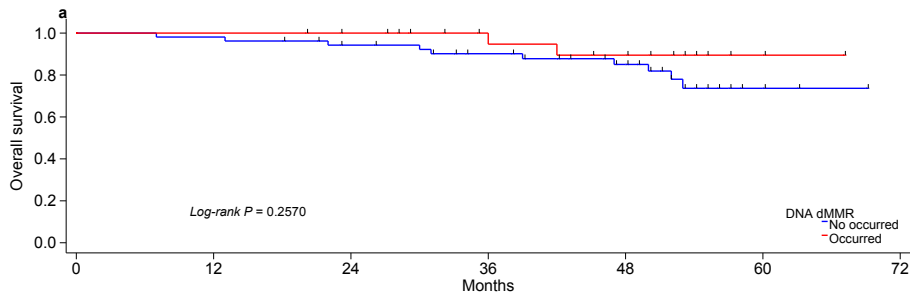

**Number at risk**

|             |    |    |    |    |    |   |   |
|-------------|----|----|----|----|----|---|---|
| No occurred | 53 | 52 | 47 | 39 | 30 | 6 | 1 |
| Occurred    | 27 | 27 | 25 | 19 | 15 | 2 | 0 |

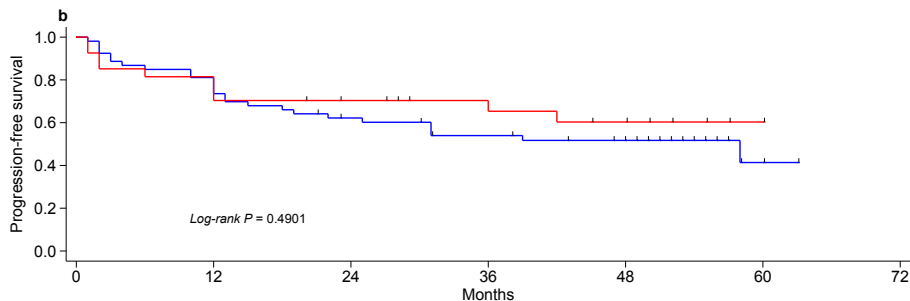

**Number at risk**

|             |    |    |    |    |    |   |   |
|-------------|----|----|----|----|----|---|---|
| No occurred | 53 | 43 | 31 | 25 | 21 | 2 | 0 |
| Occurred    | 27 | 22 | 17 | 14 | 10 | 1 | 0 |

Supplement: Supplementary file 6 [file CAM4-8-2705-s006.pdf]
