# Supplementary material for: Prognostic implications of a molecular classifier derived from whole‐exome sequencing in nasopharyngeal carcinoma
Source: Cancer Med. 2019 Apr 5;8(6):2705–16. doi: 10.1002/cam4.2146 (PMC6558473; doi:10.1002/cam4.2146)
Supplement: Supplementary file 13 [file CAM4-8-2705-s013.docx]

**Supplementary Text**

**Title:**

**Prognostic implications of a molecular classifier derived from whole-exome sequencing in nasopharyngeal carcinoma**

**Methods**

**DNA extraction**

Genomic DNA was extracted from formalin-fixed paraffin embedded tissues using the GeneRead DNA FFPE Kit (Qiagen, Hilden, Germany) following the manufacturer’s instructions. Control DNA was extracted from peripheral blood lymphocytes with QIAamp DNA Blood Mini Kit (Qiagen). Genomic DNA was analyzed on 1% agarose gels for quality control. The quantity of genomic DNA was assessed using the Qubit fluorescence detector (Life Technologies) before library preparation.

**Data processing**

Primary NPC WES data from our cohort and another three cohorts were comprehensively analyzed following the criteria below. The generated sequencing reads were checked and aligned to the human genome (Human GRCH37) with Burrows–Wheeler Aligner (BWA, 0.7.12-r1039) using default parameters ([1](#_ENREF_1)). Picard tool (v1.130, http://broadinstitute.github.io/picard/) was used to sort *bam* files and mark PCR duplications. Somatic SNVs were generated by the combination of MuTect ([2](#_ENREF_2)) (v1.1.7) and VarScan ([3](#_ENREF_3)) (v2.3.9), and short indels (≤ 50 bp) were discovered by the combination of Pindel ([4](#_ENREF_4)) (0.2.5a8) and VarScan ([3](#_ENREF_3)). Both SNVs and indels were annotated by ANNOVAR ([5](#_ENREF_5)). Eventually, SNVs and indels were further filtered out through the following steps: (i) if somatic SNVs were annotated outside EXONIC or SPLICING; (ii) if indels were > 50 bp; (iii) if somatic SNVs were common SNPs in a database, which is a union set of ESP6500 allelic frequency (AF) ≥ 0.015, 1000 genome AF ≥ 0.015; (iv) if somatic SNVs with the AF difference between tumor and normal were < 0.005; and (v) if SNVs with supporting reads for alteration < 3, total coverage < 30, and AF < 0.05. In-house developed scripts were applied to filter potential false positive SNVs and indels based on the sequencing quality and distribution. The following types of variants were prone to be filtered: (i) variants close to the ends of reads or in the simple repeat region; (ii) variants from reads with a relatively low mapping quality; (iii) variants from the mate-pair reads with alignments in different chromosomes; (iv) variants from reads with too many variants; (v) variants from reads with SNVs and indels simultaneously; and (vi) long-fragment substitution variants.

**Mutational signature analysis**

Base substitutes and mutational signatures were extracted from 82 NPC patients, and suggested that C > T substituted at NpCpG sites was the predominant mutation (Supplementary Fig. 5a), and that an age-related signature (COSMIC Signature 1) ([6](#_ENREF_6)) was present in the majority of NPC patients (Supplementary Fig. 5b, 80/82, 97.6%). The *APOBEC*-mediated signature (COSMIC Signatures 2+13) and defective DNA mismatch repair (dMMR) signature (COSMIC Signatures 6+15+20) were observed in 4 (4.9%) and 27 (32.9%) NPC patients, respectively (Supplementary Fig. 5b). A smoking-related signature (COSMIC Signature 4) was newly present in 4 NPC patients. The available clinical data showed that two patients had >20 years of smoking history. Distinct integrations of base substitutes and mutational signatures were observed in each individual, which were similar to a previous study ([7](#_ENREF_7)) and probably indicated inter-tumor heterogeneity.

Additionally, we analyzed associations between dMMR status and clinical outcomes in our cohort (Supplementary Fig. 6a and 6b). There were no significant correlations between dMMR status and survival, which may be partially due to the limited sample size and the imbalance in the proportion of dMMR signature in each sample. Berger *et al.* ([8](#_ENREF_8)) recently identified a prostate cancer patient harboring an unanticipated dMMR signature without a clear underlying causal somatic or germline lesions who was enrolled in a clinical trial for anti-PD-L1 immunotherapy, and he has exhibited a remarkable response to treatment. Further clinical trials investigating anti-PD-L1 antibody in patients with a dMMR signature are needed to fully test immunotherapy in NPC.

**References**

1. Li H, Durbin R. Fast and accurate long-read alignment with Burrows-Wheeler transform. Bioinformatics. 2010 Mar 1;26(5):589-95. PubMed PMID: 20080505. Pubmed Central PMCID: 2828108.

2. Cibulskis K, Lawrence MS, Carter SL, Sivachenko A, Jaffe D, Sougnez C, et al. Sensitive detection of somatic point mutations in impure and heterogeneous cancer samples. Nature biotechnology. 2013 Mar;31(3):213-9. PubMed PMID: 23396013. Pubmed Central PMCID: 3833702.

3. Koboldt DC, Zhang Q, Larson DE, Shen D, McLellan MD, Lin L, et al. VarScan 2: somatic mutation and copy number alteration discovery in cancer by exome sequencing. Genome research. 2012 Mar;22(3):568-76. PubMed PMID: 22300766. Pubmed Central PMCID: 3290792.

4. Ye K, Schulz MH, Long Q, Apweiler R, Ning Z. Pindel: a pattern growth approach to detect break points of large deletions and medium sized insertions from paired-end short reads. Bioinformatics. 2009 Nov 1;25(21):2865-71. PubMed PMID: 19561018. Pubmed Central PMCID: 2781750.

5. Wang K, Li M, Hakonarson H. ANNOVAR: functional annotation of genetic variants from high-throughput sequencing data. Nucleic acids research. 2010 Sep;38(16):e164. PubMed PMID: 20601685. Pubmed Central PMCID: 2938201.

6. Alexandrov LB, Nik-Zainal S, Wedge DC, Aparicio SA, Behjati S, Biankin AV, et al. Signatures of mutational processes in human cancer. Nature. 2013 Aug 22;500(7463):415-21. PubMed PMID: 23945592. Pubmed Central PMCID: 3776390.

7. Li YY, Chung GT, Lui VW, To KF, Ma BB, Chow C, et al. Exome and genome sequencing of nasopharynx cancer identifies NF-kappaB pathway activating mutations. Nature communications. 2017 Jan 18;8:14121. PubMed PMID: 28098136. Pubmed Central PMCID: 5253631 received research grant and serves the advisory board from Novartis, Hong Kong.

8. Zehir A, Benayed R, Shah RH, Syed A, Middha S, Kim HR, et al. Mutational landscape of metastatic cancer revealed from prospective clinical sequencing of 10,000 patients. Nature medicine. 2017 Jun;23(6):703-13. PubMed PMID: 28481359. Pubmed Central PMCID: 5461196.
